# Trans-scale thermal signaling in biological systems

**DOI:** 10.1093/jb/mvad053

**Published:** 2023-07-18

**Authors:** Madoka Suzuki, Chujie Liu, Kotaro Oyama, Toshiko Yamazawa

**Affiliations:** Institute for Protein Research, Osaka University, 3-2 Yamadaoka, Suita, Osaka 565-0871, Japan; Institute for Protein Research, Osaka University, 3-2 Yamadaoka, Suita, Osaka 565-0871, Japan; Department of Biological Sciences, Graduate School of Science, Osaka University, 1-1, Machikaneyama-cho, Toyonaka, Osaka 560-0043, Japan; Foundational Quantum Technology Research Directorate, National Institutes for Quantum Science and Technology, 1233 Watanukimachi, Takasaki-shi, Gunma 370-1292, Japan; Core Research Facilities, The Jikei University School of Medicine, 3-25-8 Nishi-Shimbashi, Minato-ku, Tokyo 105-8461, Japan

**Keywords:** ATPase, fluorescence microscopy, heat-induced calcium release, microheating, type 1 ryanodine receptor. *Abbreviations*: [Ca^2+^]_i_, intracellular Ca^2+^ concentration; CICR, Ca^2+^-induced Ca^2+^ release; ER, endoplasmic reticulum; FDB, flexor digitorum brevis; HEK293 cell, human embryonic kidney 293 cell; HICR, heat-induced Ca^2+^ release; IP_3_R, inositol 1,4,5-trisphosphate receptor; MH, malignant hyperthermia; RCC, rapid cooling contracture; RyR1, type 1 ryanodine receptor; SERCA, sarco/endoplasmic reticulum Ca^2+^-ATPase; SR, sarcoplasmic reticulum; TRP, transient receptor potential; WT, wild type

## Abstract

Biochemical reactions in cells serve as the endogenous source of heat, maintaining a constant body temperature. This process requires proper control; otherwise, serious consequences can arise due to the unwanted but unavoidable responses of biological systems to heat. This review aims to present a range of responses to heat in biological systems across various spatial scales. We begin by examining the impaired thermogenesis of malignant hyperthermia in model mice and skeletal muscle cells, demonstrating that the progression of this disease is caused by a positive feedback loop between thermally driven Ca^2+^ signaling and thermogenesis at the subcellular scale. After we explore thermally driven force generation in both muscle and non-muscle cells, we illustrate how *in vitro* assays using purified proteins can reveal the heat-responsive properties of proteins and protein assemblies. Building on these experimental findings, we propose the concept of ‘trans-scale thermal signaling’.

## Graphical Abstract

**Graphical Abstract:**
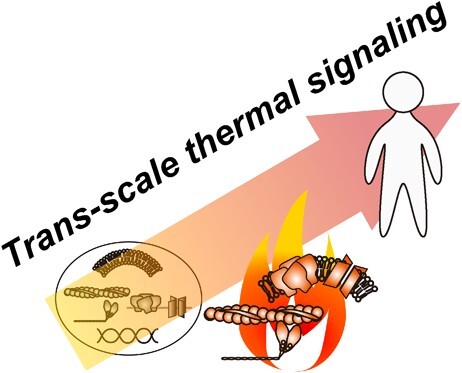

Humans maintain a constant body temperature by adapting to external thermal stimuli *(**1**)*. Due to the human body's limited capacity to store heat in cold environments, thermogenesis plays a vital role in adaptation. Two types of thermogenesis exist: shivering and non-shivering. Shivering thermogenesis involves involuntary and rhythmic contractions of skeletal muscles, serving as an initial response to cold environments, akin to an emergency evacuation. Shivering also occurs during fever episodes caused by infections and contributes to elevating the body temperature. Non-shivering thermogenesis is another thermogenic response primarily caused by sympathetically mediated metabolic surges. Brown adipose tissue, which consists of brown adipocytes, serves as a major organ responsible for non-shivering heat production, thereby maintaining body temperature in small animals like rodents and infants *(**2**,**3**)*, where heat diffusion from the body to the environment is significant due to a relatively large body surface-to-volume ratio. When thermogenesis is upregulated without proper control, serious consequences, such as fever and heat stroke can occur.

This review begins with skeletal muscle as a specific example of one of the primary sources of thermogenesis in humans and other mammals. We introduce a fatal disease known as malignant hyperthermia (MH; MIM# 145600) and highlight how it arises due to impaired thermal management at the subcellular scale. Subsequently, we explore studies conducted on model mice and individual skeletal muscle cells to investigate MH. Furthermore, we delve into the elucidation of the disease mechanism at the cellular and molecular scales. Next, we discuss the broad consequences of microscopic temperature gradients and their temporal changes resulting from the heat flow generated by local heat sources within cells. We explore a range of cellular responses, including alterations in morphology and intracellular Ca^2+^ signaling in muscle and neuronal cells. Furthermore, we explain the molecular intricacies underlying these cellular responses to demonstrate how the thermal modulation of biochemical reactions at the subcellular scale can lead to global changes within cells. Lastly, drawing from experimental observations of diverse heat-induced responses across multiple spatial scales, encompassing organisms, cells, protein assemblies and biomolecules, we propose the concept of ‘trans-scale thermal signaling’ in biological systems.

## Disease-Associated Mutations in the Type 1 Ryanodine Receptor

**Fig. 1:**
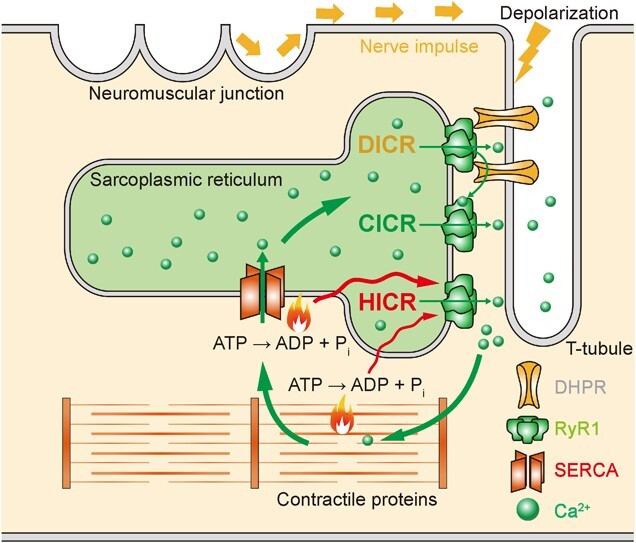
**Schematic illustration of the Ca** ^**2+**^ **flow pathway related to the type 1 ryanodine receptor in skeletal muscle cells.** When the neuromuscular junction receives a neurotransmitter such as acetylcholine, the dihydropyridine receptor (DHPR), a type of voltage-dependent Ca^2+^ channel, is activated by the depolarization of the transverse tubule (T-tubule) membrane. The direct interaction between DHPRs and the type 1 ryanodine receptors (RyR1s) converts the signal of depolarization into Ca^2+^ release from the sarcoplasmic reticulum through RyR1, which is termed depolarization-induced Ca^2+^ release (DICR). Then, Ca^2+^-induced Ca^2+^ release (CICR) is induced, where the opening of RyR1 is followed by the rapid release of Ca^2+^ from the sarcoplasmic reticulum into the cytoplasm. Muscle contraction and Ca^2+^ uptake from the cytosol into the lumen of SR, which involve contractile proteins (such as actin and myosin) and sarco/endoplasmic reticulum Ca^2+^-ATPase (SERCA), respectively, are accompanied by ATP hydrolysis and thermogenesis. These reactions in skeletal muscle are proposed as the heat sources that trigger heat-induced Ca^2+^ release (HICR) through RyR1. Wavy arrows, heat conduction.

Skeletal muscle contracts when depolarization is transmitted to the dihydropyridine receptor in the T-tubule, which is followed by the release of Ca^2+^ from the sarcoplasmic reticulum (SR) through the type 1 ryanodine receptor (RyR1) embedded in the SR membrane (Fig. 1). This process is known as excitation–contraction coupling. RyR1 is a tetramer, and each monomer consists of approximately 5,000 amino acids. RyR1 functions as a large Ca^2+^ release channel with properties of Ca^2+^-induced Ca^2+^ release (CICR) *(**4*–*6**)*. Genetic mutations in the *RYR1* gene have been linked to severe muscle diseases, including MH. Analyses of the *RYR1* gene in patients with MH and related muscle diseases have identified more than 300 different mutations located throughout the molecule, including those in the N-terminal, central and C-terminal regions *(**7**,**8**)*.

MH is triggered by inhalational anesthetics, such as halothane and isoflurane, as well as depolarizing muscle relaxants. Common symptoms of MH include an elevated body temperature reaching up to approximately 42°C and heightened skeletal muscle contractions. Mutations in RyR1 associated with MH are believed to result in significant Ca^2+^ release from the SR, primarily due to the acceleration of CICR. For example, we utilized the heterologous expression system in human embryonic kidney 293 (HEK293) cells to investigate the effects of specific mutations. We reported that the Y523S mutation in the N-terminal region and the R2508C mutation in the central region resulted in extremely severe phenotypes. These mutations were found to significantly reduce the stability of the closed state of the channel, leading to the leakage of Ca^2+^ from the SR *(**9*–*11**)*. Additionally, there have been reports linking MH mutations in the *RYR1* gene to certain cases of heat stroke. Heat stroke is a medical emergency characterized by high body temperature and altered mental status, typically triggered by exercise or exposure to extreme environmental heat.

## MH Model Mice

To gain a comprehensive understanding of the mechanisms underlying MH in humans, valid animal models are essential. Since the early 2000s, researchers have employed knock-in/knock-out mouse models carrying RyR1 mutations corresponding to human MH mutations. Several strains of MH model mice with mutations in the *RYR1* gene have been successfully generated. These include knock-in mice for R163C-RyR1 and Y523S-RyR1 in the N-terminal region, as well as G2435R-RyR1 in the central region. In these mutant mice, administration of isoflurane anesthesia induces an increase in body temperature, making them valuable models for studying MH *(**12*–*15**)*.

**Fig. 2:**
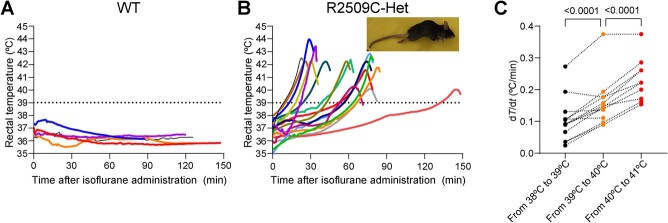
**Acceleration of an increase in temperature during malignant hyperthermia in R2509C mice.** (**A, B**) Changes in rectal temperature of WT (*A*) and R2509C (*B*) mice upon exposure to 1.5–2.0 vol% isoflurane. *n* = 5 and 15 in WT and R2509C mice, respectively (shown in different colors). *Inset*, a photograph of the R2509C-RyR1 heterozygous mouse, which responded with full body contractions as reflected in the arching of the back and extension of the legs. (C) Shift of the rate of rectal temperature elevation at the early (from 38 °C to 39 °C), middle (from 39 °C to 40 °C), and late (from 40 °C to 41 °C) stages. Statistical significance was determined by a paired *t* test. Early vs. middle, *n* = 15 and *P* = 1.4 × 10^−5^; middle vs late, *n* = 12 and *P* = 1.7 × 10^−5^. Figures are reproduced with modifications from Yamazawa et al. *(**16**)*.

Based on the findings obtained from the heterologous expression system in HEK293 cells, we proceeded to create a novel MH model mouse (R2509C-RyR1) using the CRISPR/Cas9 system. This model involved introducing a mutation (*R2509C*) in the *RYR1* gene that corresponds to the human R2508C mutation. Homozygous R2509C-RyR1 mice were found to die in early embryonic stage, whereas heterozygous R2509C-RyR1 mice exhibited normal growth and fertility comparable to wild type (WT) mice *(**16**)*. However, when exposed to isoflurane anesthesia, R2509C-RyR1 heterozygous mice exhibited a rapid increase in body temperature, surpassing 40°C, along with muscle rigidity, ultimately resulting in their demise (Fig. 2). The temperature profile of R2509C-RyR1 heterozygous mice showed a gradual rise initially followed by an accelerated rate of increase (Figs. 2B and C), culminating in a spike at approximately 39°C. These findings suggest the presence of an amplification mechanism contributing to temperature elevation during MH. In contrast, WT mice displayed no temperature elevation when exposed to isoflurane anesthesia (Fig. 2A).

In this study, we additionally verified that exposure to isoflurane led to an increase in intracellular Ca^2+^ concentration ([Ca^2+^]_i_) in enzymatically isolated single flexor digitorum brevis (FDB) muscle cells obtained from R2509C RyR1 heterozygous mice. However, no such increase in [Ca^2+^]_i_ was observed in FDB muscle cells from WT mice *(**16**)*. Then, the next question would be: is MH a phenomenon that occurs only at the animal level, or can it be reproduced at the cellular level?

## Measurement of Cellular Temperature in Isolated Single Skeletal Muscle Cells

**Fig. 3:**
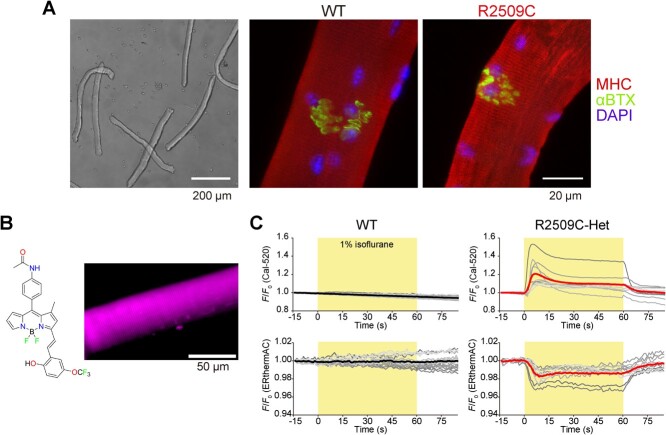
**Fluorescence imaging of [Ca** ^**2+**^ **]**_**i**_ **and temperature of the sarcoplasmic reticulum in flexor digitorum brevis muscle fibers.** (**A**) Phase contrast (*left*) and fluorescence (*center* and *right*) images of flexor digitorum brevis (FDB) muscles isolated from WT (*center*) or R2509C (*left* and *right*) mice. The fluorescence images show immunostained myosin heavy chains (MHC), nicotinic acetylcholine receptors stained with α-bungarotoxin (αBTX), and nuclei stained with 4′,6-diamidino-2-phenylindole (DAPI). (**B**) Chemical structure of ERthermAC (*left*), and a fluorescence image of FDB fiber isolated from R2509C mice following staining with ERthermAC. (**C**) Time course of changes in the relative fluorescence intensity of Cal-520 (*top*) and ERthermAC (*bottom*) in WT (*left*) and R2509C-Het (*right*) cells. Gray lines represent individual cells. Thick colored lines represent average responses. Figures are reproduced with modifications from Kriszt et al. and Tsuboi et al. *(**19**,**22**)*.

To investigate whether cellular temperature increases during MH, we examined the intracellular temperature in conjunction with [Ca^2+^]_i_ following the application of isoflurane (Fig. 3). The sarco/endoplasmic reticulum Ca^2+^-ATPase (SERCA), which is considered the heat source during MH, is located at the SR membrane; hence, the SR is presumed to be the organelle of intracellular heat source (Fig. 1) *(**17**,**18**)*. The thermogenesis by SERCA is aggravated by enhanced Ca^2+^ leakage through RyR1 mutants. Therefore, we utilized ERthermAC, a temperature-sensitive fluorescent dye that selectively targets the endoplasmic reticulum (ER) and SR *(**19**)*, to perform ER/SR-targeted thermometry in living cells (Fig. 3B). The fluorescence emission of ERthermAC can be optically distinguished from that of the fluorescent Ca^2+^ indicator Cal-520. This allows for quantitative imaging of both SR temperature and [Ca^2+^]_i_ in individual cells under a fluorescence microscope *(**20**,**21**)*. In our experiment, the fluorescence intensity of Cal-520 was increased, whereas that of ERthermAC was decreased by the application of 1% isoflurane to R2509C RyR1 heterozygous FDB cells (Fig. 3C) *(**22**)*. Note that the fluorescence intensity of ERthermAC decreased in response to an increase in temperature (as in references *(**19*–*21**)*. Based on the simultaneous measurements of [Ca^2+^]_i_ and cellular temperature, we reached the conclusion that the increase in [Ca^2+^]_i_ observed upon the application of isoflurane is indeed associated with an increase in cellular temperature during MH.

## Thermally Driven Ca^2+^ Signaling in Cells

Living organisms sense temperature in various ways. For example, temperature affects liquid–liquid phase separation in plants *(**23**)* and RNA editing in octopuses *(**24**)*. In mammals, temperature-sensitive transient receptor potential (TRP) channels play a crucial role in sensing of the absolute temperature of our bodies and the surrounding environment. The discoveries of these channels by David Julius and Ardem Patapoutian were honored with the 2021 Nobel Prize in Physiology or Medicine *(**25**,**26**)*. The open probabilities of thermosensitive TRP channels change within a narrow range of the temperature, and responsive temperature range is specific to the respective channels; *e.g.* > 42°C for TRPV1, ~ 27°C to 42°C for TRPV4 and < 25°C TRPM8 *(**27**)*. In all of the contexts mentioned above, temperature is regarded as a macroscopic parameter that changes relatively slowly.

We need to remind ourselves that the open probability of ion channels is modulated as well by a relatively fast temperature change, *i.e.* a heat flow. One prominent example is a rapid cooling contracture (RCC), where the rapid cooling induces [Ca^2+^]_i_ increases and contractions in muscles *(**28**)*. RyR1, a major Ca^2+^-release channel in skeletal muscles as introduced in sections above, also responds to cold stimuli *(**29**)* and releases Ca^2+^ during RCC *(**30**)*. However, it appears that the MH-related RyR1 mutants release Ca^2+^ during the progression of MH, as evidenced by Figures 2 and 3. If this holds true, it suggests that the heat sensitivity of RyR1 mutants is elevated compared to their WT counterparts. In other words, RyR1 mutants may be more susceptible to Ca^2+^ release in response to temperature increases.

**Fig. 4:**
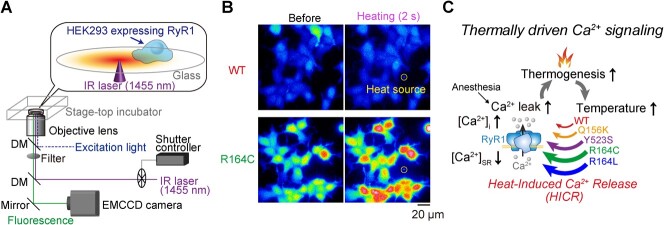
**Investigation of the heat sensitivities of RyR1 MH mutants.** (**A**) Illustration depicting the experimental design. (**B**) Fluorescence images capturing heat-induced Ca^2+^ bursts observed in fluo-4-loaded HEK293 cells expressing wild type (WT) RyR1 or R164C mutants. (**C**) The positive feedback loop at the SR membrane, involving local temperature elevation due to cellular thermogenesis by SERCA and thermally driven Ca^2+^ release via RyR1 mutants, is closed by heat-induced Ca^2+^ release (HICR), leading to the progression of malignant hyperthermia. Figures have been reproduced with modifications from Oyama et al. *(**21**)***.**

We directly tested this hypothesis by subjecting HEK293 cells expressing rabbit skeletal RyR1 WT or mutant variants to 2-s optical heat pulses. We then observed the resulting Ca^2+^ dynamics using the fluorescent Ca^2+^ indicator fluo-4 under a fluorescence microscope (Fig. 4) *(**21**)*. Our results demonstrated that the hypothesis is correct. A smaller amplitude of the heat pulse, Δ*T*, could induce a [Ca^2+^]_i_ increase in mutant cells than in WT cells, indicating an abnormal heat sensitivity of the mutants. Especially, at the physiological temperature (36°C), [Ca^2+^]_i_ increase was observed in R164C(L) mutant cells as a response to Δ*T* lower than 1.4°C, which was smaller than the body temperature increase during the progression of MH. We named the novel finding that heat can be the agonist of RyR1 ‘heat-induced Ca^2+^ release (HICR)’ (Fig. 1). HICR was also confirmed in skeletal muscles isolated from R2509C RyR1 heterozygous mice, where the [Ca^2+^]_i_ of the muscles is increased by the optical heating above 40°C. Therefore, it was proposed that a positive feedback loop at the SR membrane between local temperature increases following cellular thermogenesis by SERCA and thermally driven Ca^2+^ release via RyR1 mutants accelerates the progression of MH (Fig. 4C).

At the end of this section, we consider a combination of Ca^2+^ channels and SERCA activities as a heat-sensing system within living cells. The thermogenesis of SERCA is a byproduct of its ATPase activity, which is known to accelerate with rising temperatures. Additionally, the temperature sensitivity of Ca^2+^ channels further adds to the complexity of this system. The interplay between these factors governing Ca^2+^ signaling can lead to unpredictable Ca^2+^ responses to heat stimuli. Examples are as follows.

In a study by Tseeb et al., a thermally driven Ca^2+^ response was investigated. The researchers applied a brief 2-s heat pulse to HeLa cells using the optical heat pulse method, which involved utilizing a metal aggregate that absorbed a focused 1,064 nm laser beam *(**31**)*. During the heating period, there was a decrease in [Ca^2+^]_i_ due to heat-activated Ca^2+^ uptake by SERCA from the cytoplasm into the ER lumen. However, immediately after the heating ended, [Ca^2+^]_i_ exhibited a significant increase, surpassing the level observed prior to heating, which is referred to as ‘Ca^2+^ overshoot’. It was established that the dominant Ca^2+^-release channel responsible for the Ca^2+^ overshoot was not the temperature-sensitive TRP channels, but rather the inositol 1,4,5-trisphosphate receptor (IP_3_R) located within the ER membrane. Surprisingly, the heat pulse required to induce the Ca^2+^ burst had a remarkably low threshold amplitude of just 0.2°C when the experimental temperature was set to the body temperature of 37°C. Additionally, similar Ca^2+^ responses were observed not only in the other cell line, HEK293 cells *(**21**)*, but also in normal human fetal lung fibroblast WI-38 cells *(**32**)*. Considering the cell type-independent molecular mechanism, such a highly heat-sensing system based on the combination of IP_3_R and SERCA may be intrinsic to the cells composing our bodies.

## Thermally Driven Force Generation in Cells

Ca^2+^ signaling in cardiac and skeletal muscles regulates their force generation. During the initiation of cardiac muscle contractions, depolarization of the outer membrane triggers Ca^2+^ influx through the voltage-dependent L-type Ca^2+^ channel. This is then followed by Ca^2+^ release from the SR. In contrast, in skeletal muscles, depolarization directly triggers Ca^2+^ release from the SR (Fig. 1). The transient [Ca^2+^]_i_ increase, known as the Ca^2+^ transient, plays a critical role in initiating force generation in the myofibrils.

Temperature exerts complex effects on multiple biochemical reactions and influences the performance of muscles *(**33**)*. In cardiac muscles, for example, heating leads to a decrease in the amplitude of Ca^2+^ transients, which in turn results in lower pressure in the beating heart at higher temperatures. Conversely, the maximal force generation of Ca^2+^-activated myofibrils increases with temperature. Additionally, the Ca^2+^ sensitivity of myofibrils is enhanced as temperature rises, meaning that myofibrils initiate force generation at lower [Ca^2+^]_i_ when exposed to higher temperatures. These heat sensitivities of myofibrils might alleviate the decrease in pressure of the beating heart when body temperature is increased.

Surprisingly, recent studies have revealed that muscle contraction can be activated by heat independently of Ca^2+^ signaling. Our research has shown that brief optical heat pulses ranging from 0.2 to 0.5 s can induce contraction in rat cardiac myocytes, even in the absence of any changes in [Ca^2+^]_i_*(**34**)*. Similar heat-induced contractions in skeletal myotubes (C2C12) were observed by optical heating of nanoparticles loaded in the myotubes *(**35**,**36**)*. In both cases, a myosin inhibitor, blebbistatin, blocked the heat-induced contractions, indicating that these contractions were caused by actin-myosin interactions as in the physiological Ca^2+^-induced contractions.

Before delving into the molecular details of thermally driven force generation based on actin-myosin interactions, it is worth briefly mentioning other examples of the thermally driven motility in non-muscle cells. Firstly, ‘thermotaxis’ is a directional migration that occurs due to spatial temperature gradients. It has been observed in, *e.g. Dictyostelium discoideum (**37**)* and rabbit sperm cells *(**38**)*. Secondly, a steep spatial temperature gradient induces unequal cell division in grasshopper spermatocyte *(**39**)*. It also results in an asymmetric force generation on the actin cortex at the plasma membrane of spherical HeLa cells, causing a directional extension of the plasma membrane, or blebbing, toward the warmer side *(**40**)*. Thirdly, rat hippocampus neurons responded to optical heating. Elongation of neurites and a change in the morphology of the growth cone were induced within 60 s upon heating *(**41**)*. Heat-enhanced actin-myosin interactions, polymerization of microtubules, and their sliding by motor proteins were all demonstrated to be involved in the heat-induced neurite elongation.

## Molecular Assemblies behind the Thermally Driven Force Generations

**Fig. 5:**
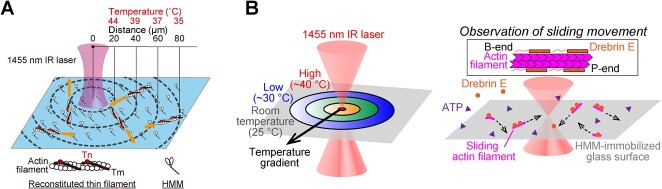
**Schematic illustrations of the *in vitro* motility assays that are combined with the IR laser-based local heating.** (**A**) Reconstituted thin filaments from actin filaments and troponin (Tn)-tropomyosin (Tm) complexes interacted with heavy meromyosin (HMM; α-chymotrypsin proteolytic fragment of myosin II) attached to the glass surface. Local temperature within the field of view of the fluorescence microscopy was directly increased by a focused IR laser beam (λ = 1,455 nm), which elicits thin filament movements (as shown by orange arrows). (**B**) Reconstituted filaments from actin filaments and drebrin E interacted with HMM attached to the glass surface. In both assays, the sliding velocity was dependent on the distance from the heat source, *i.e.* the temperature. Figures are reproduced with modifications from Ishii et al. and Kubota et al. *(**42**,**44**)*.

The bottom-up approach is a powerful strategy to identify heat-sensing biomolecules or molecular assemblies. For instance, the heat-induced muscle contraction introduced in the previous section *(**34*–*36**)* can be reproduced in the *in vitro* motility assay system using only four types of muscle proteins: actin, myosin, tropomyosin, and troponin *(**42**)*. The *in vitro* motility assay involves the use of purified myosin molecules or their enzymatically digested fragments that are attached to a glass surface. Subsequently, the sliding movement of fluorescently labeled actin filaments or reconstructed filaments, which consist of actin filaments and actin filament-binding proteins such as the troponin-tropomyosin complex, is observed under a fluorescence microscope in the presence of ATP (Fig. 5).

**Fig. 6:**
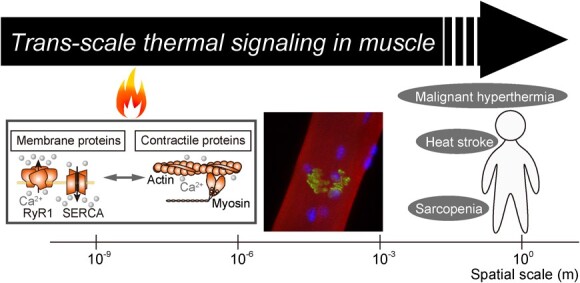
**Schematic illustration of the proposed trans-scale thermal signaling in muscle, as a representative of biological systems.** Diseases, such as malignant hyperthermia, are caused at the scale of organisms when the thermal management at the molecular scale is impaired.

Ishii et al. conducted an experiment in which they combined the *in vitro* motility assay using reconstituted muscle thin filaments with the optical heating method (Fig. 5A). These reconstituted thin filaments were immobile at low [Ca^2+^] (~10^−9^ M), representing the relaxed state of muscle. However, the researchers discovered that when subjected to optical heating for 2 s, the previously immobile filaments exhibited sliding *(**42**)*. This result is a direct demonstration at the molecular level of the thermally driven and Ca^2+^-independent actin-myosin force production that has been observed at the cellular level. Surprisingly, heating to physiological temperature (~37°C) induced relatively slow sliding of reconstituted cardiac thin filaments (~30% velocity compared to the maximal Ca^2+^-activated filaments). One probable mechanism is a thermally induced partial dissociation of the troponin-tropomyosin complex from actin filaments that has been reported previously from birefringence measurements at > ~ 45°C *(**43**)*. These results indicate that the actin-myosin interaction in cardiac muscles is, at least partially, activated even in the relaxed state at body temperature.

A similar heat-sensing mechanism has also been identified in a non-muscular force-generating system involving actin and myosin and their regulatory protein, drebrin E. Drebrin E is primarily expressed in nerve cells during embryonic development and acts to inhibit actin–myosin force generation. In the *in vitro* motility assay composed of actin filament and drebrin E (Fig. 5B), Kubota et al. reported that the inhibition of filament sliding was weakened during the optical heating, so that the sliding was elicited *(**44**)*. They confirmed that fluorescently labeled drebrin E molecules remained bound to actin filaments during heating. This observation led to the proposal of a mechanism similar to the assembly of actin filaments and the troponin–tropomyosin complex, where a part of individual filamentous drebrin E molecules dissociates from actin filaments at relatively higher temperatures. In summary, the interactions between actin and myosin, as well as their regulation by biomolecules, are not thermally robust even at physiological temperatures, but they are sensitive to small changes in temperature.

Lastly, in this section, we will introduce thermally induced hyperactivity in molecular motors. Although the motor activity can be reversibly regulated at the single molecule level *(**45**,**46**)*, prolonged exposure to hyperthermic conditions can lead to a decrease in their activity, primarily caused by thermal damage. For example, myosin becomes inactive within 1 min at 50°C. However, Kato et al. demonstrated in the *in vitro* motility assay that the sliding velocity of actin filaments was continuously increased up to and above 60°C if the period of time of the optical heat pulses was as short as 1/16 s *(**47**)*. Kawaguchi and Ishiwata likewise reported that the gliding velocity of microtubules over kinesins increased along with the Arrhenius equation in the range between 15°C and 50°C *(**48**)*. They observed no breaks in the relationship, indicating that kinesin ATPase is potentially activated above the physiological point.

These hyperactive molecular motors, observed in the *in vitro* systems, are capable of functioning at temperatures above normal body temperature in living cells. Our study demonstrated that the active transport of endosomes/lysosomes along microtubules, facilitated by kinesins and/or dyneins, was significantly enhanced when subjected to heating up to 47°C for 1 s *(**49**)*. Interestingly, the temperature coefficient *Q*_10_ in cells was determined to be 2.6. This value exceeds the previously determined *Q*_10_ value of approximately 2 in the *in vitro* motility assay. This discrepancy suggests the presence of other biomolecules that regulate motor activities, thereby influencing the observed heat-sensitive active transport on microtubules.

## Concluding Remarks

Force and heat generation are major physiological functions of skeletal muscles. However, our understanding of the relationship between these two functions is primarily biased toward the process of ‘force generation → thermogenesis’, particularly during physical exercises or shivering thermogenesis. The other direction, ‘thermogenesis → force generation’, is considered the relationship via the body temperature in between, *i.e.* ‘thermogenesis → body temperature rise → elevation of actin–myosin ATPase rate and force generation’. Our limited understanding of the latter may be caused by our common practice of thermogenesis and its consequences at the cellular scale: ‘The heat power released as a byproduct of biochemical reactions by individual proteins or cells is very low. However, the assembly of 37 trillion cells can maintain the human body's temperature at 37°C. This macroscopic function of maintaining the body temperature is the most important and the only function of heat.’

Such a consideration is mostly correct. The heat released from a heat source diffuses through the boundary between the heat source and the external environment. Therefore, although it is possible to maintain body temperature at the scale of larger organisms, which have a smaller surface/volume ratio, the heat released from individual chemical reactions or cells is apparently lost by diffusion in the surrounding aqueous environment, leaving no meaningful results for intracellular processes or cells. However, can we apply this general concept to any of the largely diverse biological processes?

In this review, we discussed the challenges associated with the general concept, highlighting specific examples that have been experimentally demonstrated by both ourselves and other researchers. Conventional tools are primarily designed to treat temperature as a macroscopic parameter. However, addressing the intricacies of heat flow in living cells at the microscopic scale requires unconventional methods capable of manipulation and measurement. Therefore, many of the studies introduced here have employed novel methods of microscopic heating and temperature measurement developed relatively recently *(**50**)*. We began with MH, the result of an out-of-control cycle of thermogenesis and heat-responsive Ca^2+^ signaling in skeletal muscles. These experiments suggest the importance of proper thermal management at the subcellular scale. Following the thermally driven Ca^2+^ signaling were the thermally driven force generations, *i.e.* the processes of ‘thermogenesis→ heat flow→ force generation’, in muscle and non-muscle cells. The *in vitro* motility assays demonstrated a powerful bottom-up approach to examining their molecular mechanisms directly.

With such a variety of thermally driven biochemical processes in biological systems, we have been inspired by the possibility that even the heat flow from cellular thermogenesis may result locally in Ca^2+^ signaling, force generation, and acceleration of active transport *(**51**)*. The examples may include any other chemo-mechanical coupling, such as the acceleration of transcription, or biochemical processes, such as liquid–liquid phase separation. The heat seems to function as a mediator of intracellular signaling. Therefore, we previously proposed to label the hypothetical contribution of endogenous intracellular heat flow as ‘thermal signaling’ *(**52**)*. The heat, unlike materials, such as Ca^2+^ or ATP, emanates from biochemical reactions, traverses biological membranes and can have substantial impacts on organisms. Given its ability to traverse multiple spatial scales, we refer to it as ‘trans-scale thermal signaling’ (Fig. 6) *(**53**)*.

There are several issues that were not addressed in this review. It is imperative to investigate thermogenesis and thermal signaling in biological molecules at atomic resolution *(**53**)*. Additionally, it is important to understand the concept of ‘temperature’ as determined by luminescence thermometry at the microscopic or smaller scales within cells *(**54**)*. Furthermore, the mechanisms of heat flow within the intracellular space need to be explored *(**52*,*55*–*60**)*. Lastly, it is crucial to determine the extent to which significant temperature changes can be expected from biochemical reactions occurring in cells *(**52**,**55**,**57**)*. For those interested in delving into these matters, we recommend consulting the respective references.
